# Persecutory Delusion in Parkinson’s Disease: At the Crossroad Between Psychosis and Dementia

**DOI:** 10.1192/j.eurpsy.2025.1725

**Published:** 2025-08-26

**Authors:** K. Mahfoudh, A. Hakiri, H. Khiari, A. Jaoua, I. Besbes, A. Bouallagui, G. Amri, R. Ghachem

**Affiliations:** 1 Department B, Razi Hospital, Manouba, Tunisia

## Abstract

**Introduction:**

Patients with Parkinson disease (PD) may present psychiatric manifestations like delusions and hallucinations which can significantly impact their quality of life. These psychotic symptoms may also occur during neuro-cognitive decline which poses a diagnostic challenge.

**Objectives:**

This case study aims to investigate the clinical overlap between psychosis and cognitive decline in Parkinson’s disease, focusing on the diagnostic difficulties in differentiating these psychiatric symptoms.

**Methods:**

We report a single case of a patient admitted to the psychiatric department “B” of Razi Hospital, who exhibited symptoms of both psychosis and cognitive decline. We also conducted a literature review on Pubmed using the following keywords: Parkinson disease, Dementia, Delirium, psychosis.

**Results:**

Mr. T.J, a 64-year-old man with a 10-year history of PD, was treated with levodopa, amantadine, and pramipexole. He developed depression a year ago, managed with fluoxetine. Recently, he was admitted to the psychiatric ward due to severe behavioral disturbances, including hetero-aggressiveness towards his wife.

Psychiatric assessment revealed a delusional syndrome with themes of jealousy and persecution towards his wife, accompanied by a hallucinatory syndrome primarily characterized by auditory hallucinations that appeared 2 years ago.

A neurological examination identified an amnesic syndrome that preceded the onset of delusions, raising concerns about the differential diagnosis between psychosis and dementia in the context of PD with the added consideration of the iatrogenic effects of his Parkinson’s treatment.

In collaboration with neurologists, we adjusted the treatment regimen by tapering off amantadine and pramipexole while maintaining levodopa. We introduced 5 mg of olanzapine, which led to a favorable clinical response. After two weeks, Mr. T.J was considered stable enough for discharge. Improvements included a reduction in delusional ideation and a decrease in the frequency and severity of auditory hallucinations.

**Conclusions:**

This case highlights the complexity of managing psychiatric symptoms in Parkinson’s disease, particularly the challenge of differentiating between psychosis and dementia. The positive outcome following medication adjustments underscores the importance of a multidisciplinary approach in addressing both motor and psychiatric symptoms in PD patients.

**Disclosure of Interest:**

None Declared

